# Genetic structure of the Mon-Khmer speaking groups and their affinity to the neighbouring Tai populations in Northern Thailand

**DOI:** 10.1186/1471-2156-12-56

**Published:** 2011-06-15

**Authors:** Wibhu Kutanan, Jatupol Kampuansai, Silvia Fuselli, Supaporn Nakbunlung, Mark Seielstad, Giorgio Bertorelle, Daoroong Kangwanpong

**Affiliations:** 1 Department of Biology, Faculty of Science, Chiang Mai University, Chiang Mai 50200, Thailand; 2 Dipartimento di Biologia ed Evoluzione, Universita di Ferrara, Ferrara, Italy; 3 Department of Sociology and Anthropology, Faculty of Social Sciences, Chiang Mai University, Chiang Mai 50200, Thailand; 4 Department of Laboratory Medicine & Institute for Human Genetics, University of California San Francisco, California, 94143, USA

## Abstract

**Background:**

The Mon-Khmer speaking peoples inhabited northern Thailand before the arrival of the Tai speaking people from southern China in the thirteenth century A.D. Historical and anthropological evidence suggests a close relationship between the Mon-Khmer groups and the present day majority northern Thai groups. In this study, mitochondrial and Y-chromosomal DNA polymorphisms in more than 800 volunteers from eight Mon-Khmer and ten Tai speaking populations were investigated to estimate the degree of genetic divergence between these major linguistic groups and their internal structure.

**Results:**

A large fraction of genetic variation is observed within populations (about 80% and 90% for mtDNA and the Y-chromosome, respectively). The genetic divergence between populations is much higher in Mon-Khmer than in Tai speaking groups, especially at the paternally inherited markers. The two major linguistic groups are genetically distinct, but only for a marginal fraction (1 to 2%) of the total genetic variation. Genetic distances between populations correlate with their linguistic differences, whereas the geographic distance does not explain the genetic divergence pattern.

**Conclusions:**

The Mon-Khmer speaking populations in northern Thailand exhibited the genetic divergence among each other and also when compared to Tai speaking peoples. The different drift effects and the post-marital residence patterns between the two linguistic groups are the explanation for a small but significant fraction of the genetic variation pattern within and between them.

## Background

Northern Thailand consists of many plains and mountains, usually stretching in a north-south direction. Most of this wide area is covered by forests and fertile land that was occupied by large numbers of people since prehistoric times [1].

Today, the Tai speaking peoples represent the major linguistic group in Northern Thailand, but archaeological evidence reveals that this area was occupied by Mon-Khmer speaking groups such as Mlabri, H'tin, Lawa, and Mon since the prehistoric period [1]. The first kingdom-level development was the Mon of Haripunchai (750 A.D.-1300 A.D.), and the earliest datable stone inscriptions (from 1218 to 1219 A.D.) mentioned Lawa as another local population [2]. The decline of the Mon kingdom occurred in the thirteenth century when a Tai group migrated from south and south-east China. They conquered the native populations on their southern route until they reached the northern part of what is now Thailand. Some Mon groups fled south to central Thailand, but many remained in this area under the Tai rulers [1]. These people were later assimilated and acculturated by Tai migrants [3]. The Mon ethnic group is cited in many historical records of the civilizations of northern Thailand, suggesting that this specific Mon-Khmer speaking population played an important role during the Tai immigration and for the establishment of the present day populations in northern Thailand. In general, archaeological and historical evidence suggests a close relationship between modern Mon-Khmer and Tai speaking groups in this area, but their biological affinity has not yet been established.

**Figure 1:**
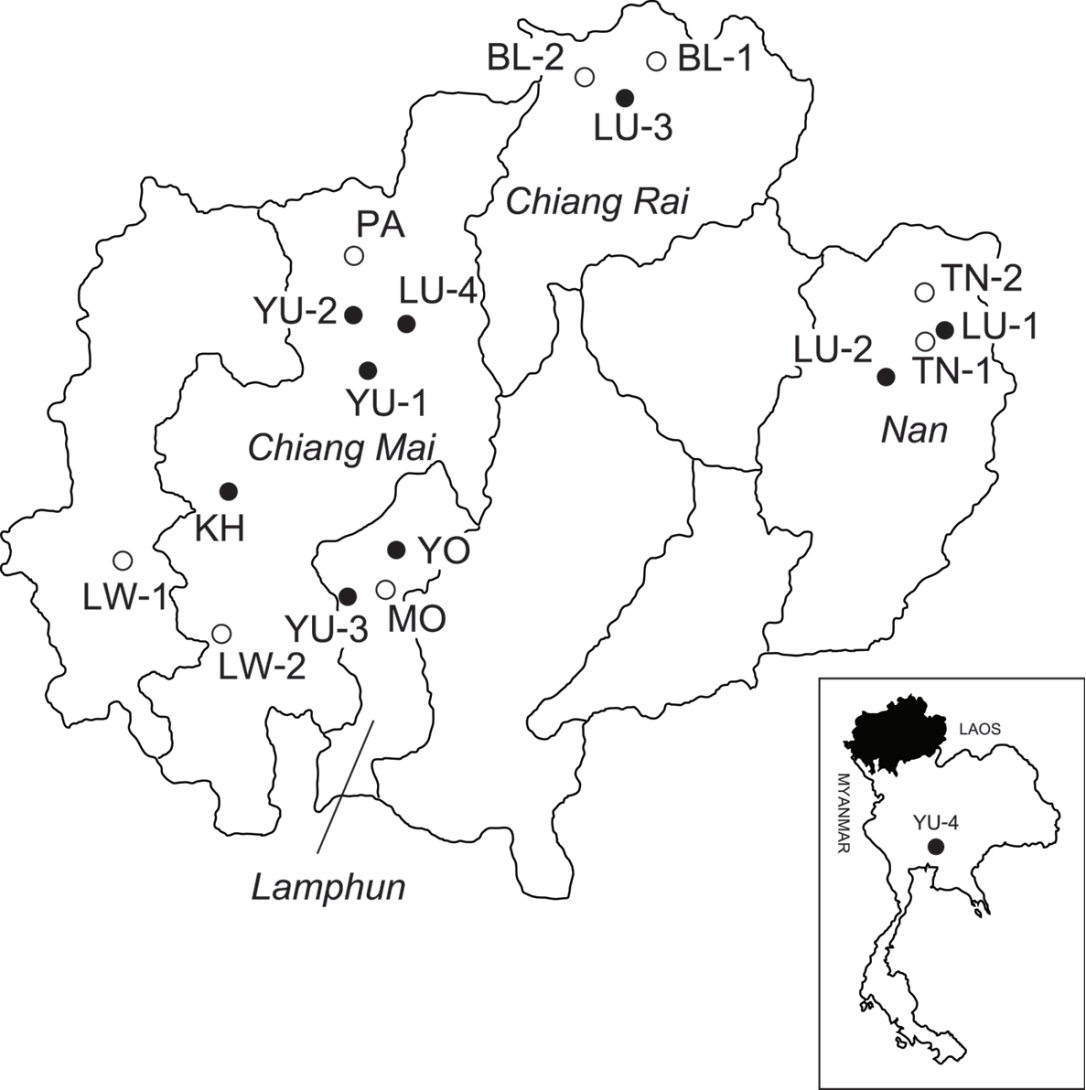
**Geographic distribution of population samples**. Filled circles: Tai linguistic subfamily; Empty circles: Mon-Khmer linguistic subfamily.

Genetic variation of the Y-chromosome and the mitochondrial genome has been used widely in population genetic studies. As they are transmitted uniparentally, through either paternal or maternal lineages, the population history can be reconstructed separately for each gender. These data can be used, therefore, to identify unequal contributions between males and females in migration rates, polygamy patterns and specific rules of post-marital residence [4-6]. Here we analyse patterns of genetic variation of seventeen short tandem repeats loci on the Y-chromosome (Y-STRs) and 336 bp of the control region of the mitochondrial DNA (mtDNA), to investigate the genetic structure and the relationships within and among different Mon-Khmer and Tai populations in northern Thailand (Table 1 and Figure 1). The factors affecting the genetic patterns are discussed.

**Table 1:** Basic indices of genetic diversity within populations

							Population diversity indices								
	Code	Latitude	Longitude	Sample size			Y-STRs				mtDNA				
		(°N)	(°E)	Male	Female	Total	No.of haplotypes	*h *^a^	S.D.	MSD^b^	No.of haplotypes	*h*	S.D.	*π (10^2^)*^c^	S.D.
Linguistic affiliation															
(Family, Subfamily)															
**Austroasiatic, Mon-Khmer**															
Mon	MO	98°53 '	18°31'	15	26	41	13	0.98	0.03	1.56	16	0.92	0.02	2.18	1.16
Lawa1	LW1	97°56 '	18°23 '	25	21	46	15	0.95	0.02	1.28	25	0.96	0.01	1.90	1.02
Lawa2	LW2	98°20'	18°08 '	25	25	50	18	0.95	0.03	1.68	15	0.91	0.02	1.93	1.03
Paluang	PA	99°09'	19°56'	23	28	51	11	0.90	0.04	2.28	20	0.92	0.02	1.65	0.90
Blang1	BL1	99°52'	20°25'	18	20	38	17	0.99	0.02	1.76	25	0.98	0.01	2.26	1.20
Blang2	BL2	99°50'	20°08'	22	23	45	20	0.99	0.02	2.04	28	0.97	0.01	2.33	1.23
H'tin1 (Mal)	TN1	100°55'	19°08'	20	17	37	10	0.93	0.03	1.07	12	0.74	0.06	1.60	0.88
H'tin2 (Pray)	TN2	100°54'	19°19'	20	18	38	16	0.98	0.02	2.20	9	0.69	0.07	1.90	1.02
								**0.96**		**1.73**		**0.89**		**1.97**	
Linguistic affiliation															
(Family, Subfamily)															
**Tai Kadai, Tai**															
Yuan1	YU1	98°59'	19°00'	20	19	39	18	0.99	0.02	2.10	26	0.97	0.01	2.15	1.14
Yuan2	YU2	98°59'	19°11'	25	25	50	21	0.98	0.02	2.68	30	0.97	0.01	2.26	1.19
Yuan3	YU3	98°45'	18°24'	26	24	50	20	0.97	0.02	1.93	28	0.97	0.01	2.22	1.17
Yuan4	YU4	100°53'	14°33'	21	23	44	20	1.00	0.02	2.34	21	0.95	0.01	2.17	1.15
Lue1	LU1	100°56'	19°09'	25	26	51	22	0.99	0.01	2.22	23	0.92	0.03	1.95	1.04
Lue2	LU2	100°47'	19°05'	21	23	44	17	0.98	0.02	2.32	14	0.88	0.03	2.10	1.12
Lue3	LU3	99°53'	20°26'	26	24	50	25	1.00	0.01	1.84	39	0.99	0.01	2.21	1.17
Lue4	LU4	99°07'	18°52'	24	22	46	20	0.98	0.02	2.56	19	0.93	0.02	1.91	1.02
Khuen	KH	98°51'	18°38'	29	31	60	25	0.99	0.01	2.70	31	0.97	0.01	2.51	1.31
Yong	YO	98°56'	18°24'	31	31	62	26	0.99	0.01	2.02	31	0.97	0.01	2.22	1.16
								**0.99**		**2.27**		**0.95**		**2.17**	

^a ^h, haplotype diversity; ^b ^MSD, mean squared allele size differences averaged over loci; ^c ^*π*, nucleotide diversity; SD, standard deviation

## Results

### Genetic variation within populations

In total, 321 Y-STRs haplotypes, and 277 mtDNA haplotypes were observed among 416 males, and among 842 individuals, respectively. Statistics describing the levels of genetic variation within populations, and their mean in Mon-Khmer and Tai groups, are reported in Table 1. Haplotype diversity is always greater than 90% for both male and female lineages, with only two relevant exceptions (69% and 74%) in the H'tin populations for mtDNA sequences. Average pairwise distances between individuals are more variable among populations, but sampling errors are large. On the average, Mon-Khmer populations appear less variable than Tai populations, but statistical significance with a non parametric test (Mann-Whitney U-test) is reached only for the mean pair-wise difference among Y-chromosomes.

### Genetic differences between populations and major linguistic groups

**Figure 2:**
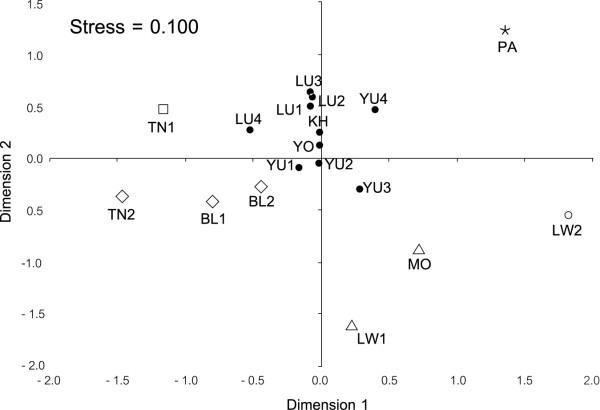
**Multidimensional scaling scatter plot based on the Slatkin's linerization *Rst *matrix, applies to Y-chromosome**. Filled circles: Tai linguistic subfamily; Empty symbols: Mon-Khmer linguistic subfamily with different shapes indicating BAPS cluster's membership.

**Figure 3:**
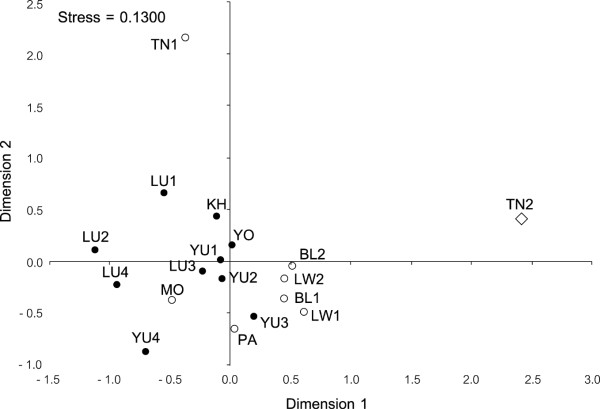
**Multidimensional scaling scatter plot based on the pairwise *Fst *matrix, applies to mtDNA**. Filled circles: Tai linguistic subfamily; Empty symbols: Mon-Khmer linguistic subfamily with different shapes indicating BAPS cluster's membership.

Around 97% and 77% of haplotypes are private (found in a single population) at Y-chromosome and mtDNA, respectively. Haplotype sharing is therefore very limited, though higher for both markers among Tai than among Mon-Khmer populations (6 vs 4 for Y-chromosome and 27 vs 14 for mtDNA). The two major groups, Mon-Khmer and Tai, share only one Y-chromosome haplotype and 22 mtDNA haplotypes. The MDS plot of the paternal lineages (Figure 2) shows that Tai speaking populations are confined in a central cloud, whereas most of the Mon-Khmer populations are scattered around it. What seems evident in the figure is confirmed by the almost six times larger *F_st _*value among Mon-Khmer populations compared to the *F_st _*value among Tai populations (Table 2). The maternal marker shows a different pattern (Figure 3). Only two Mon-Khmer populations, H'tin-Mal (TN1) and H'tin-Prai (TN2), can be considered to be highly divergent samples. The Tai populations and the remaining Mon-Khmer samples are genetically close, though the value of Dimension 1 in the MDS, with a single exception represented by the Mon (MO), can still be used to discriminate between the two groups.

**Table 2:** Analysis of molecular variance (AMOVA)

			% of variance					
	No. of groups	No. of populations	Within populations	Among populations within groups	Among groups	*F_st_*	*F_sc_*	*F_ct_*
**Y chromosome**								
All samples	1	18	80.8	19.2		0.1920*		
Tai	1	10	94.16	5.84		0.0584*		
Mon-Khmer	1	8	65.75	34.25		0.3425*		
Tai/Mon-Khmer	2	18	79.96	17.93	2.11	0.2004*	0.1832*	0.0211
**mtDNA**								
All samples	1	18	92.8	7.2		0.0720*		
Tai	1	10	95.03	4.97		0.0497*		
Mon-Khmer	1	8	91.09	8.91		0.0892*		
Tai/Mon-Khmer	2	18	92.14	6.41	1.45	0.0786*	0.0650*	0.0145*

* *P *≤ 0.001

The AMOVA analysis was used to estimate the proportion of the genetic variation accounted for by groups defined on the basis of their linguistic affinities (Table 2). The overall *F_st _*value is higher in male (0.192, *P *<0.01) than in female (0.072, *P *<0.01) lineages.

The genetic divergence between populations, at both paternally and maternally inherited markers, is higher in the Mon-Khmer group (MO, LW1, LW2, TN1, TN2, PA, BL1, BL2) than the Tai group (YU1, YU2, YU3, YU4, LU1, LU2, LU3, LU4, KH, YO ), and this difference is larger for Y-chromosomes (Table 2). In other words, the Mon-Khmer group appears more genetically structured than the more homogeneous Tai group, and possibly with a higher sex-related bias in migratory patterns.

The proportion of genetic variation between the two linguistic groups is quite limited (around 1 to 2%). This fraction is statistically different from zero only for the much larger mtDNA data set. Thus it seems that, on average, Tai and Mon-Khmer populations are similar, or, to be more precise, only slightly more divergent than populations within each group. We note, however, that the interpretation of this hierarchical analysis is not straightforward, since the average level of population differentiation within the two linguistic groups is very different.

Finally, Bayesian analysis of population structure (BAPS) was employed to ascertain how many different groups of populations are supported by Y chromosome and mtDNA data. Despite the limited power of this approach when only one locus is analyzed, results of both markers are consistent with the MDS explorative analysis (Figure 2 and 3). In particular, the Y-chromosome data set highlights the similarity of Tai populations, all assigned to the same cluster, while Mon-Khmer populations show a considerable level of divergence, being assigned to 5 distinct clusters (Figure 2). Population structure for mtDNA is lower, and only the highly divergent sample H'tin-Prai (TN2) is separated from all the rest of the studied populations (Figure 3).

### Correlation between genetics, language, and geography

The relative impact of geographic and linguistic distances on the genetic divergence between pairs of populations were clearly different. Geography seems to have little relation to mtDNA and Y-chromosome differentiation, whereas genetic distances are significantly correlated with linguistic affiliation in language families and dialects (Table 3). The partial correlation coefficients between genetic and linguistic distances are 0.24 and 0.28 for mtDNA and Y-chromosomes, respectively, meaning that about 6-8% of the genetic variation is explained by linguistic variation. Small changes in the metrics used to define linguistic distances do not affect these results. In other words, genetic similarity does not appear to be higher when geographically closer populations are compared, but among other factors, linguistic affinity explains a moderate but significant fraction of the genetic divergence pattern.

**Table 3:** Correlation and partial correlation coefficients, *r *value and *P*-value (in parenthesis), between genetic, geographic and linguistic matrices

	Y chromosome		mtDNA	
**Matrices considered**	**Correlation coefficient (r)**	**Proportion of variance explained (r^2^)**	**Correlation coefficient (r)**	**Proportion of variance explained (r^2^)**

Dgen and Dgeo, Dlan constant	0.09	0.008	0.14	0.02
Dgen and Dlan, Dgeo constant	0.28*	0.08	0.24*	0.06

* *P *< 0.005

Dgen: Fst genetic distance

Dgeo: geographic distance

Dlan: linguistic distance

## Discussion

The main observation from our study is that the genetic divergence between populations, in the paternal lineages, is higher in the Mon-Khmer than in the Tai. This difference is probably a signature of historical and/or demographic processes combined with cultural differences in the post-marital residence patterns. A plausible explanation of our results is that Tai immigrants maintained genetic homogeneity whereas drift, during and after the Tai colonization, enhanced the genetic divergence among Mon-Khmer populations, since after the Tai invasion in the thirteenth century, the Mon-Khmer group was fragmented and some ethnic groups were exiled to rural areas [1].

Paternal and maternal lineages provide contrasting results, as has been observed frequently in human population studies [7]. Typical explanations include sex-biased post-marital residence pattern [8] and polygyny [9]. We are not aware of high levels of polygyny (implying smaller male than female effective population sizes) in Mon-Khmer, or of larger diffusion of polygyny in Mon-Khmer compared to Tai people. On the other hand, we note that our results are compatible with sex-biased post-marital residence pattern in the Mon-Khmer. Mon-Khmer populations are, in fact, patrilocal (i.e., the wife moves into her husband's house), with the exception of the H'tin (TN1 and TN2, Mal and Pray respectively) who are matrilocal. On the other hand, in the Tai populations neither strict patrilocality nor matrilocality predominates. Clear genetic traces of these cultural traditions can be found in our results: i) the genetic divergence among populations measured by Y-chromosome lineages is higher among Mon-Khmer than among Tai populations; ii) in Mon-Khmer, but not in Tai, the genetic divergence between populations is larger in the Y-chromosome compared to mtDNA lineages; iii) the H'tin populations are highly differentiated and have lower internal diversity in the mtDNA sequences. We note also that the large and independent divergence of the two H'tin populations (Mal and Pray), is possibly related to their specific history and due to their different languages. Many small groups of H'tin people had been wandering between Thailand/Laos border and resettled in Nan province of northern Thailand around 70-90 years ago [10], suggesting a large impact of the founder effect and drift in these groups.

Overall, only a small fraction of the observed genetic distances can be attributed to the Mon-Khmer/Tai distinction (between 1 to 2%). This result, which should be interpreted carefully, because population divergence patterns are very different within the Mon-Khmer and the Tai, might indicate that the original immigrants and residents in Northern Thailand were genetically similar, or that gene flow occurred after the Tai immigration. The first hypothesis, given the large cultural difference between Mon-Khmer and Tai people, seems unlikely. The second hypothesis, assuming that Tai immigrants incorporated Mon-Khmer residents [3], with the latter partially maintaining their identity, appears more likely and compatible with our results on the genetic structure within the two groups.

When linguistic differences between populations are considered in more detail, including distance measures for languages and dialects, a larger fraction of genetic variation (around 6 to 8%) can be explained by linguistic affiliation as shown in several ethnic groups [11-14], and should not be a surprise. Surprisingly, the geographic distances do not seem to play any role in favoring (when low) or reducing (when high) the migration pattern. It seems that genetic distances between the populations we analysed are affected by many factors including history, language, and post-marital residence patterns, but not simply by the geographic distance between villages.

Finally, we would like to mention the specific case of the Mon. Historical evidence indicates that the Mon had been the most developed Mon-Khmer civilization before the arrival of Tai people in the thirteenth century [1]. This fact can explain why immigrant Tai males were inclined to marry Mon females [15,16], which in turn might be related to the central position of the Mon within a cloud of Tai populations in the MDS plot based on mtDNA distances (Figure 3). Only the analysis of more markers and more individuals will possibly clarify if this pattern of genetic variation was affected by the assimilation pattern specific of the Mon population.

## Conclusions

Mon-Khmer and Tai speaking populations show a different pattern of internal genetic structure. Most of Mon-Khmer populations are highly divergent, in the paternal lineages, among each other as well as when compared to Tai populations, and two of them, the H'tin (Mal and Prai), also exhibit the same pattern in the maternal lineages. On the other hand, the Tai populations are genetically similar for both markers. These results can be explained by different drift effects in the two groups, possibly enhanced in small and fragmented populations in the Mon-Khmer, and cultural differences in the post-marital residence patterns.

## Methods

### Studied populations and DNA extraction

We studied 842 volunteers (416 males and 426 females) from 18 villages belonging to 9 ethnic groups from the northern part of Thailand (Table 1 and Figure 1). Informed consent was obtained from each subject. Information on linguistic, cultural aspects, village and individual history was obtained by interview.

Five milliliters of peripheral blood were obtained from each individual using a vacutainer coated with anticoagulant-EDTA. Total genomic DNA was extracted from whole blood sample according to a standard inorganic salting out protocol [17].

### Genotyping and sequencing

All male individuals were genotyped for 17 Y-STRs. The primers were synthesized by Applied Biosystems, USA. All loci were amplified in 5 multiplex polymerase chain reactions [18-20]:

Multiplex 1: DYS19, DYS388, and DYS390

Multiplex 2: DYS391, DYS392, and DYS393

Multiplex 3: DYS389a/b and DYS426

Multiplex 4: DYS434, DYS435, DYS436, DYS437, and DYS439

Multiplex 5: Y-GATA-A7.1, Y-GATA-A7.2, and Y-GATA-A7.10

Amplicons were separated by multi-capillary electrophoresis in an ABI3100 genetic analyzer (Applied Biosystem, Foster City, CA). Results were then analyzed by GeneMapper software v. 3.0 and 3.7 (Applied Biosystem, Foster City, CA).

MtDNA control region of eight Mon-Khmer speaking villages was amplified using published primer pairs [21]. The purified PCR products were sequenced for hypervariable region I (HVRI) with the BigDye Terminator Cycle Sequencing Kit v3.1 and ABI 3730 DNA Analyzer (Applied Biosystem, Foster City, CA). Sequencing was performed using a published set of primers [21,22]. The 336 bp at the position 16048-16383 were edited, assembled, and aligned with the revised Cambridge Reference Sequence [23] using SeqScape software v2.5 (Applied Biosystem, Foster City, CA). In addition, mtDNA sequences of the same length (336 bp) from Yuan, Lue, Yong, and Khuen were obtained from a previous study [24].

The HVR-1 sequences of all samples were submitted to GenBank (accession numbers HM634245-HM634590).

### Statistical analysis

To describe genetic diversity within populations, the number of observed haplotypes and the haplotype diversity (*h*) [25], were calculated from both genetic systems. The mean squared allele size differences averaged over loci (MSD) was computed from Y-chromosome data, and the nucleotide diversity (*π*)[26] was calculated from the mtDNA sequence data.

Pairwise genetic distance between populations were computed using *R_st _*for Y-STRs [27] and *Φ_st _*for mtDNA sequences. We consistently refer to these statistics in the text as *F_st _*statistics. Matrices of the *F_st _*were then represented in two dimensions by means of a multidimensional scaling (MDS) (STATISTICA 7.0 software package, StatSoft Inc, Padova, Italy)

The analysis of molecular variance (AMOVA) [28] was performed to quantify the genetic diversity at three hierarchical levels, namely, between members of the same population, between populations of the same group, and between groups of samples. Here, the groups were defined to encompass two linguistic subfamilies, Mon-Khmer subfamily: MO, LW1, LW2, TN1, TN2, PA, BL1, BL2 and Tai subfamily: YU1, YU2, YU3, YU4, LU1, LU2, LU3, LU4, KH, YO. The significance of the fixation indices is tested using a non-parametric permutation approach [28].

Bayesian analysis of population structure using the software BAPS version 5.2 [29-31], an approach that assigns single populations to a non-predefined number of groups, was performed to identify the likely number of homogenous groups of populations.

Geographic-, genetic-, and linguistic-distance matrices were tested for possible correlation. Matrices were compared by means of nonparametric Mantel partial correlation tests [32]. Diversity indices, genetic distances, AMOVA and Mantel tests were calculated using the software ARLEQUIN 3.11 [33]. The mean squared allele size differences from Y-chromosome STRs was calculated using Genpop on the Web [34].

Linguistic distances between pairs of populations were defined as simple dissimilarity indices on the basis of the hierarchical classification of languages reported in Ethnologue [35]. Populations speaking languages belonging to different subfamilies (i.e., Mon-Khmer and Tai) were assigned dLAN of 3, different languages within subfamilies dLAN of 2 or 1, depending on their level of dissimilarity, clearly differentiated dialect dLAN of 1 (i.e., Lawa (LW1-2), Blang (BL1-2), and H'tin (TN1-2)), otherwise dLAN of 0 was assigned to populations speaking the same language (Table 4). Two additional linguistic distance matrices were constructed, the first increasing dLAN between subfamilies from 3 to 4, and the second increasing dLAN between different populations speaking the same language (i.e., LU 1-4 and YU 1-4) from 0 to 1.

**Table 4:** Linguistic distance matrix

	Austroasiatic, Mon-Khmer								Tai-Kadai, Tai		
	MO	LW1	LW2	PA	BL(1,2)	TN1	TN2	KH	LU	YU	YO
**MO**	*								(1,2,3,4)	(1,2,3,4)	
**LW1**	**2**	*									
**LW2**	**2**	**1**	*								
**PA**	**2**	**1**	**1**	*							
**BL(1-2)**	**2**	**1**	**1**	**1**	*						
**TN1**	**2**	**2**	**2**	**2**	**2**	*					
**TN2**	**2**	**2**	**2**	**2**	**2**	**1**	*				
KH	3	3	3	3	3	3	3	*			
LU(1,2,3,4)	3	3	3	3	3	3	3	1	*		
YU(1,2,3,4)	3	3	3	3	3	3	3	2	2	*	
YO	3	3	3	3	3	3	3	2	2	2	*

Bold letter: Mon-Khmer speaking populations. For population names see Table 1. Samples Blang (BL) 1-2, Lue (LU) 1-4, and Yuan (YU) 1-4 have been merged in this table because their linguistic distance is 0.

## Competing interests

The authors declare that they have no competing interests.

## Authors' contributions

WK and JK contributed equally in collecting samples, molecular genetic data generating, statistical analysis, interpretation of data, and drafting the manuscript. SF participated in performing the statistical analysis and helped to draft the manuscript. SN participated in interpretation of data and discussion. MS participated in molecular genetic data generating. GB involved in revising the manuscript critically for important intellectual content. DK conceived of the study and coordination, discussed, and revised the manuscript. All authors read and approved the final manuscript.
